# The Levine laboratory and the discovery of p53

**DOI:** 10.1093/jmcb/mjz027

**Published:** 2019-08-13

**Authors:** Daniel Linzer

**Affiliations:** Research Corporation for Science Advancement, Tucson, AZ, USA

**Figure 1:**
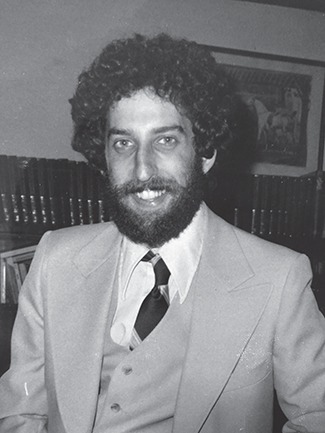
Daniel Linzer at Princeton in 1979.

As a first-year graduate student in the fall of 1976, I completed a research rotation on DNA tumor viruses in Arnie Levine's laboratory at Princeton and then decided on that research area and research group for my doctoral work. Arnie welcomed me into his laboratory with his typically infectious enthusiasm and high energy. At the time, graduate students and postdoctoral fellows in the group were working on a number of DNA viruses. Among those was simian virus 40 (SV40), and Arnie pointed me toward a set of questions on this virus's infectious cycle in monkey cells and its ability to induce tumor formation in mice. It turned out that these separate questions converged.

One question concerned the identification of proteins encoded by the very small SV40 genome. Animals with SV40-induced tumors produced antibodies that could be used to bind to and precipitate these viral proteins. SV40-infected cells could be labeled with a radioactive amino acid, the proteins extracted and mixed with the antiserum, and finally the immunoprecipitated, radioactively labeled proteins identified as bands on gels exposed to X-ray film. Angie Teresky, Arnie's long-time research associate and laboratory manager, was an expert at producing these antisera, and she provided me with sera from several tumor-bearing mice to start my analysis.

The large tumor (T) antigen had been reasonably well characterized already by the approach of tumor serum immunoprecipitation of virus-infected cell extracts. More recently, a smaller protein recognized by tumor antibodies had been discovered and was referred to as the small tumor (t) antigen. For the closely related DNA tumor virus, polyoma, a third protein had been described and called middle T antigen. The expectation in the Levine laboratory (and probably in most DNA tumor virus laboratories) was that SV40 would similarly encode a middle T antigen of ~50 kDa. So, I began a search for that presumed SV40 middle T antigen.

A second question was to identify the biochemical steps in the infection pathway by comparing cells in which SV40 infection proceeded to different extents. Previous research had shown that SV40-infected mouse cells failed to produce mature virus particles and furthermore that the number of steps in the infectious cycle completed after introduction of SV40 into mouse fibroblasts differed from the number of steps completed after introduction of the virus into a set of tumor cells known as embryonal carcinomas. Why would the infection fail in mouse cells but go to completion in monkey cells, and why would the infection proceed to two different degrees in non-tumor mouse fibroblasts compared to these mouse tumor cells?

The third question was how the SV40 tumor antigens, especially large T antigen, interfered with normal cellular regulation to convert a non-tumor cell into a tumor cell. At the time, much of the excitement around DNA tumor viruses was that they might serve as a molecular window into the transformation of a well-behaved cell into a cancer cell. But how? Surely, the SV40 tumor antigens must interact with the cellular growth regulatory machinery, yet it remained to be determined if T antigen, and perhaps other tumor antigens, altered the expression of certain cellular genes, stimulated a higher level of activity of certain enzymes, or interfered with the function of certain proteins.

Some 40 years later, these questions might in hindsight seem straightforward to attack. At the time, of course, our (or maybe it was just my) understanding of the complexities of the SV40-infected cell and the toolbox of analytical techniques that could be deployed were limited. In any event, I struggled to make headway. I do not recall any sense of frustration, though, primarily because Arnie was such an effective cheerleader. It did not matter if the results of an experiment were negative; his response was to praise what did work (`Hey, that is a great looking gel!’). And, if I (or someone else in the laboratory) happened on a potentially interesting result, the response might be to get a group together to go celebrate with a beer at the student center (where, more often than not, Arnie would intend to treat, only to find his wallet empty). That constant support and enthusiasm, coupled with the excitement of kicking around ideas in an informal manner in the laboratory or in Arnie's office or at the student center at any hour, made it fun to work in the laboratory.

It therefore came as a shock to me that one day when I brought to Arnie a dripping wet film fresh out of the darkroom that his reaction was `I don't believe it.’ Not, `I don't believe it! Wow, this is great!’ Rather, `I don't believe it’ with the tone saying `this can't be right.’ Arnie, the master motivator, recognized the potential importance of the result, but by reacting as he did, he lit a fire under me to demonstrate that this result was real.

That film was eventually part of our 1979 publication on what became known as p53. It showed that antibodies in the tumor serum that Angie had provided to me were able to react with both the expected large T antigen in SV40-infected cells and another protein of about 50 kDa in the SV40-infected fibroblasts. The surprise was that this other protein could not be the hypothesized SV40 middle T antigen, because I was also able to detect it in uninfected embryonal carcinoma cells. This was confusing. Why was this protein present in SV40-infected fibroblasts but not detected in uninfected fibroblasts, yet it was present in both infected and uninfected embryonal carcinoma cells? How could a tumor serum that supposedly differed from normal mouse serum only by the presence of antibodies against viral proteins include antibodies against a protein present in uninfected cells? Well, maybe the protein in the uninfected cancer cells was just coincidentally the same size as the protein in infected fibroblasts. I had to figure out if these two proteins were indeed the same.

Fortunately, a graduate student down the hall in Marc Kirschner's laboratory, Don Cleveland, had just invented a peptide mapping technique that I could use to answer that question. By breaking the proteins into smaller pieces, it would be possible to compare all the pieces of the two proteins to see if they were identical in size or not, which would then reveal if the two parent proteins were the same. Don guided me in this procedure, and it worked like a charm. The result was that I could show that the proteins were indeed the same, so this protein—despite it only being detected in fibroblasts after virus infection and despite it being recognized by tumor antibodies—must be a cellular, not a viral, protein.

The detection in uninfected embryonal carcinoma cells of what we now know to be p53 demonstrated that the antiserum contained antibodies against this protein. Therefore, the failure to detect p53 in uninfected fibroblasts indicated that levels of this protein were constitutively lower in these mouse cells compared to the cancer cells. It then followed that SV40 infection was causing p53 to accumulate at higher levels than normally present in fibroblasts. It could be that SV40, and presumably T antigen, was upregulating synthesis of this cellular protein, reducing its degradation rate, or both. One way that T antigen might limit degradation would be if it bound to and stabilized p53. We were able to test that with a panel of monoclonal antibodies against T antigen; each of these antibodies (which recognized T antigen but not p53) was able to retrieve from infected cell extracts both T antigen and p53, so p53 must have `come along for the ride’ by binding to T antigen. The finding that T antigen physically interacted with p53 raised the exciting possibility that in some way p53 was a key player in the tumorigenic process initiated by SV40.

The interaction of T antigen and p53 could also explain the presence of antibodies against p53 in the tumor serum. If tumors contained high levels of T antigen in a complex with p53, the mouse immune system might then recognize p53 in this complex as foreign. However, T antigen could not be the only way to alter p53 levels. The embryonal carcinoma cells without any SV40 proteins must have activated a mechanism other than T antigen stabilization of p53 to bring about higher levels of this protein. It would be several years later that Moshe Oren in Arnie's group would unravel that mystery, showing that these cancer cells expressed a mutant form of p53 that accumulated at high levels.

One of the remarkable outcomes of this research was that Arnie made a big bet on this line of investigation. He could have continued to oversee a laboratory that was working on a range of viral systems, a surer way to make steady contributions, but more and more he turned the attention of the laboratory to p53. That was a risky play, but then the ability to see ahead to what direction would likely turn out to have the most impact is a characteristic of the best scientists and that certainly includes Arnie. His enthusiasm and support for students and postdocs as they struggle to make progress, his ability to offer guidance from broad knowledge and experience without overly proscribing what experiments should be done, and his nose for following the path of the most important science even if that might lead to a more challenging set of problems to solve, are qualities that made it incredibly enjoyable to be in his research group.

**Figure 2:**
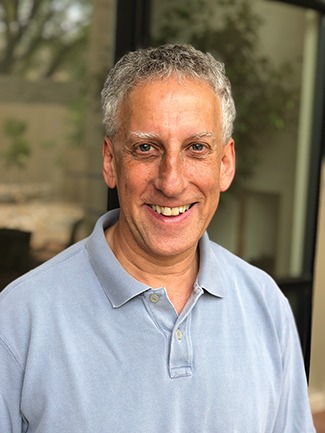
Daniel Linzer in 2018, with a slight difference over 40 years.

